# Application of Intracytoplasmic Morphologically Selected Sperm Injection (IMSI) on a Teratozoospermic Patient and Its Effect on the In-Vitro Fertilization (IVF) Outcome

**DOI:** 10.7759/cureus.53268

**Published:** 2024-01-31

**Authors:** Gauri Gajabe, Akash More, Jarul Shrivastava, Namrata Choudhary, Shilpa Dutta, Krushnali S Kadu, Ritesh Jadhav

**Affiliations:** 1 Clinical Embryology, Datta Meghe Institute of Higher Education & Research, Wardha, IND

**Keywords:** introcytoplasmic sperm injection (icsi), intracytoplasmatic morphologically selected sperm injection (imsi), infertility female, poor ovarian reserve, teratozoospermia

## Abstract

Infertility, defined as the inability to conceive after 12 months of unprotected sexual activity, affects millions globally. Approximately 80% of cases have identifiable causes, including endometriosis, tubal obstruction, ovulatory dysfunction, and male sperm abnormalities. Lifestyle factors, such as smoking and obesity, also impact fertility. Sperm morphology, a key factor in male infertility, often presents as teratozoospermia, with defects in the head, midpiece, or tail. Poor ovarian reserve, indicated by low anti-mullerine hormone (AMH) and antra-follicular count (AFC) values, contributes to female infertility, often exacerbated by age-related factors. Elevated follicle-stimulating hormone (FSH) levels further diminish oocyte quantity and quality.

Intracytoplasmic Sperm Injection (ICSI), a micromanipulation technique aiding infertile couples, may face challenges in detecting subtle sperm morphology defects. Advanced methods like Motile Sperm Organelle Morphological Examination (MSOME) and Intracytoplasmic Morphologically Selected Sperm Injection (IMSI) under high magnification enhance sperm selection accuracy. We present the case of a 36-year-old woman and her 42-year-old husband who sought assistance after seven years of infertility. Previous Intrauterine injection (IUI) and ICSI attempts failed due to the wife's low ovarian reserve and elevated FSH, compounded by the husband's teratozoospermia. Their earlier In-Vitro Fertilization (IVF) experience yielded a single poor-quality oocyte, hindering blastocyst formation. Investigations revealed the wife's poor AFC, AMH of 0.033ng/ml, and FSH at 24IU/L. Her medical history included hypertension and gallbladder removal. The husband exhibited 98% defective sperm, devoid of a substance abuse history. The wife's family had a polycystic ovarian syndrome (PCOS) history, and her low AMH and AFC yielded only three poor-quality oocytes during the current assessment. Oocytes were retrieved, and sperm were selected with the help of IMSI. After ICSI, the patient successfully conceived.

## Introduction

Infertility occurs when a couple is unable to conceive after 12 months of unprotected sexual activity. About 80% of couples have identifiable causes for their infertility, while the remaining 20% have an unknown cause of infertility. The most well-known identifiable causes of infertility are endometriosis, tubal obstruction, ovulatory dysfunction, and elevated parameters in male sperm. Numerous factors, such as smoking, alcohol consumption, obesity, etc., have an impact on fertility [1]. Infertility affects more than 20 million men and 37 million women worldwide and is a severe health concern [2]. Another factor contributing to male infertility is sperm morphology. Many morphological complex changes occur during spermatogenesis, resulting in sperm structure defects. Various defects in sperm structure, like head defects, mid-piece, and tail defects, occur. More than 96% of defective sperm are referred to as teratozoospermia [3]. One of the causes of female infertility is poor ovarian reserve characterized by a very low AMH value and a poor AFC value. It might be related to the age of the patient. Elevated levels of the hormone FSH reduce both the quantity and quality of oocytes. It is important to assess the ovarian reserve to achieve success [4].

Intracytoplasmic sperm injection (ICSI) is a gamete micromanipulator that can help infertile patients with elevated sperm parameters. However, in some rare cases, defective sperm morphology may not be visible with ICSI. In such cases, highly magnified sperm selection techniques help observe the proper morphology of the sperm. It is possible to select sperm with minimal defects for ICSI using the motile sperm organelle morphological examination technique, which has been re-examined since 2001, combined with high magnification [5]. The high power magnification of the IMSI inverted light microscope results in a magnification of x1500 [6].

For ICSI, a male gamete selected under high magnification is used. This technique is known as intracytoplasmic morphologically selected sperm injection (IMSI). A successful ICSI outcome and excellent predictive accuracy can be achieved in teratozoospermic patients by selecting sperm under high magnification [7].

## Case presentation

Patient information

The patient is a 36-year-old woman who visited our clinic with her 42-year-old husband. She married at the age of 29 and has subsequently faced infertility problems for the past seven years. She has had one IUI and one ICSI failure. She had low ovarian reserve and an elevated FSH level, and her 42-year-old husband, who has teratozoospermia, has 98% of their sperm defective. Before visiting our infertility clinic, a couple went to another IVF clinic. Ovum pickup was scheduled, and only one oocyte of MI grade was retrieved. Due to the poor quality and large perivitelline space in her oocyte, ICSI was performed, but no blastocyst was formed. After investigating FSH, AMH, and AFC levels, we found that she had a poor AFC and an AMH of 0.033 ng/ml with an elevated FSH level of 24 IU/L.

The husband's semen parameters were abnormal. He has a 98% defective sperm, or teratozoospermia, and had no prior history of drug consumption.

The female patient had a family history of PCOS. She had previously complained about gaining weight, had a low AMH value, poor AFC, and elevated hormonal levels. She also had a history of hypertension and had undergone gallbladder removal surgery in the past. Due to low AMH values and poor AFC counts, only three oocytes of very poor quality were retrieved. Low-quality oocytes prevent blast formation, and they degenerate. The male had no prior history of alcohol, tobacco, or drug use.

Medical history

The patient had a history of hypertension and poor ovarian reserve. She was prescribed 4mg of perindopril once a day for hypertension. She had no history of hypothyroidism. The male patient's only medical history was teratozoospermia.

Clinical findings

The general examination of the female patient was normal. Her blood sugar level was average. She had a low AMH, i.e. 0.33 ng/ml. Her AFC was poor, i.e. 3. She had a blood pressure of 135/88 mmHg, indicating hypertension. Her FSH level had increased to 24 mlU/ml, which implies poor oocyte quality. Due to a decrease in AMH value and poor antral follicular count, it was concluded that the patient suffers from poor ovarian reserve. Her husband’s semen analysis was done in which his sperm count was 75 million/ml, motility was 60%, and defects were 98%, indicating teratozoospermia.

Treatment

**Figure 1:**
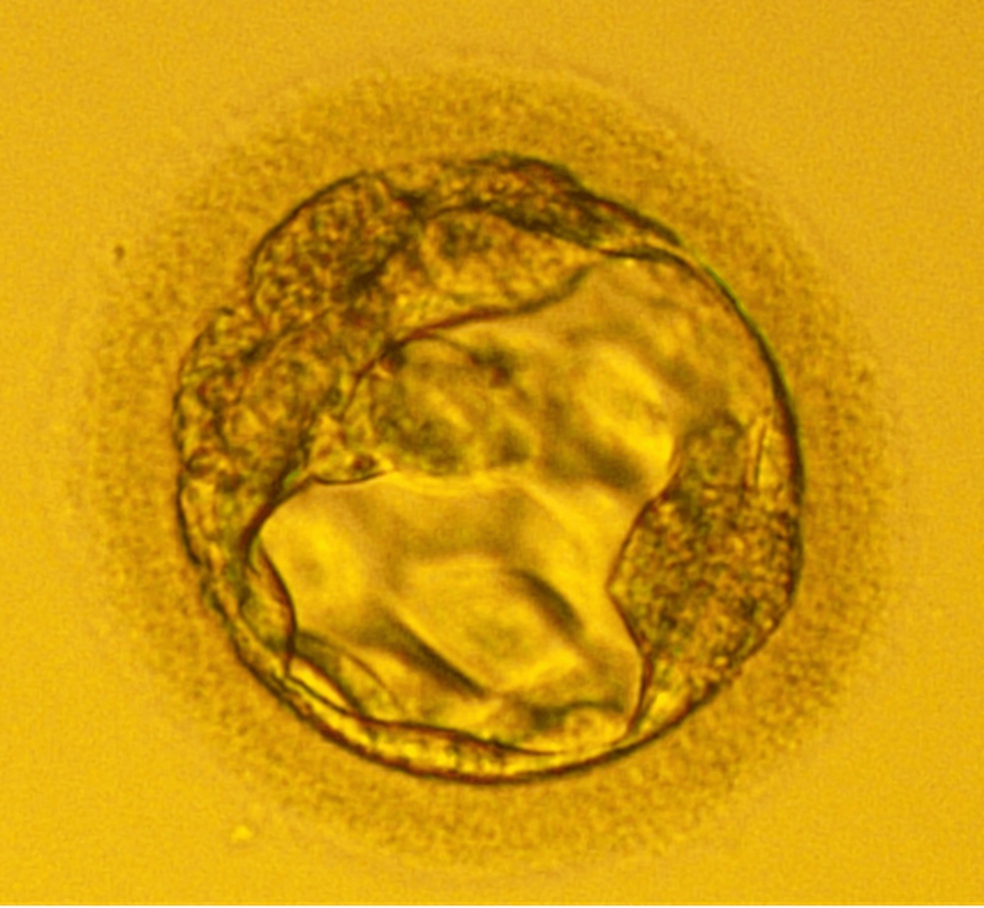
Day 5 poor quality embryo of grade 3BB

The patient visited our IVF clinic in March 2022 with poor ovarian reserve. Since the husband’s count was 75 million/ml and the defect was 98%, we could not proceed with IUI because we required normal morphology spermatozoa for ICSI. That was only possible in IMSI. Therefore, we scheduled ovum pick-up (OPU); before OPU, we started a short antagonist protocol for the patient. We started human menopausal gonadotropins (HMG) for one day with a daily dose of 300mg for the first three days and 450mg for the rest of the seven days. HMG induces follicle maturation and speeds up corpus luteum development. Gonadotropin hormone (GH) was administered (2 units) for 8 days. We also started cetrorelix once a day for four days. The ovulation stimulation treatment is controlled by cetrorelix, which delays premature ovulation. The patient was triggered 36 hours before the OPU with human chorionic gonadotropins (hCG). Two oocytes were obtained, one MI from the left and one MII from the right. Then, a processed semen sample was taken, and sperms were selected with the help of IMSI prior to ICSI. ICSI was successfully done. The fertilised embryos were cultured for five days. On day 5, we examined the embryo and observed that the blast of Grade 3BB (Blastocoel, represented by '3,' filled the blastocyst. The inner cell mass ('B') was arranged severally and loosely packed, while another 'B' represented the trophectoderm, organized in several cells and forming a loose epithelium.") was formed, as shown in Figure 1. We cryopreserved the embryo. After one month, we scheduled the embryo transfer. We started oestradiol 2mg twice daily and progesterone 150 mg once a day through injections 20 days prior to embryo transfer to enhance the endometrial thickness. After 20 days, embryo transfer was done.

Follow-up

Follow-up was conducted after 14 days of embryo transfer. A blood sample (10ml) was taken to test beta-human chorionic gonadotropin (β-hCG). The value of β-hCG was 657mlU/mL. The patient, who had poor ovarian reserve, and her husband, with defective sperm (Teratozoospermia) that was selected with the help of IMSI, had successfully conceived a pregnancy. After becoming pregnant, the patient started with multivitamins like calcium, iron, folic acid, and vitamin D. 

## Discussion

Around 80% of couples struggling with infertility have identifiable causes; the remaining 20% face the issue of unexplained infertility. Some well-known factors contributing to infertility include endometriosis, tubal obstruction, ovulatory dysfunction, and abnormalities in sperm parameters [1,8]. Lifestyle factors such as smoking, alcohol consumption, and obesity also play a significant role in influencing fertility. Sperm morphology, a crucial aspect of male fertility, can contribute to infertility. Spermatogenesis, the process of sperm development, involves complex changes that can result in structural defects [9]. These defects may appear as head, mid-piece, or tail abnormalities, resulting in teratozoospermia. To effectively manage male infertility, sperm morphology concerns must be recognised and addressed.

Female infertility can be attributed to various factors, one of which is poor ovarian reserve. This condition is characterized by a low AMH value and a poor AFC [10]. Age is a significant factor, and poor ovarian reserve is often associated with elevated levels of FSH, leading to reduced quantity and quality of oocytes [4]. Assessing ovarian reserve is crucial for determining the chances of success in fertility treatments. ICSI has emerged as a powerful tool in assisted reproductive technology to address infertility associated with elevated sperm parameters [11]. However, in some cases, traditional ICSI may not reveal defects in sperm morphology adequately [12]. This limitation has led to the development of highly magnified sperm selection techniques to observe and select sperm with optimal morphology. One such technique is the MSOME, which was introduced in 2001. Combining high magnification with traditional examination methods enables the detailed assessment of sperm morphology, allowing for the selection of sperm with minimal defects for ICSI. Using an inverted light microscope with a magnification of x1500, known as IMSI, has further refined this approach [13]. The IMSI technique involves selecting a male gamete under high magnification before performing ICSI. This meticulous selection process aims to identify and use sperm with optimal morphology, which is particularly beneficial in cases of teratozoospermia [14].

## Conclusions

ICSI stands out as an innovative micromanipulation technique for infertility, especially in cases with elevated sperm parameters. However, conventional ICSI faces challenges in cases of elusive defective sperm morphology. MSOME, introduced in 2001, addresses this by meticulously assessing sperm morphology using high magnification. IMSI, an advancement of MSOME, utilises an x1500 inverted light microscope, proving instrumental in cases of teratozoospermia. IMSI's precision in selecting sperm with optimal morphology enhances the success of fertilisation, offering new possibilities in assisted reproductive technologies for couples facing infertility challenges.
